# 1-Carboxymethyl-1′-carboxylatomethyl-3,3′-[*p*-phenylenebis(oxymethylene)]dipyridinium bromide dihydrate

**DOI:** 10.1107/S1600536810037748

**Published:** 2010-09-30

**Authors:** Hong-Lei Lian, Wei-Cheng Pan

**Affiliations:** a College of Chemical Engineering, Zhengzhou University, Zhengzhou, Henan 450001, People’s Republic of China; b College of Chemical Engineering and Foods, Zhongzhou University, Zhengzhou, Henan 450044, People’s Republic of China

## Abstract

In the crystal structure of the title salt, C_22_H_21_N_2_O_6_^+^·Br^−^·2H_2_O, pairs of betaine mol­ecules are bridged by protons (the bridging proton is disordered), forming strong and symmetrical O—H⋯O hydrogen bonds, leading to an infinite chain along the *b* axis. The water mol­ecules are linked to the betaine mol­ecule and the bromide ion through O—H⋯O and O—H⋯Br inter­actions. The central ring, located on an inversion centre, makes dihedral angles of 1.2 (2)° with the outer rings. One of the carboxylic acid groups is deprotonated.

## Related literature

For a related structure, see: Zhang *et al.* (2004 ▶). 
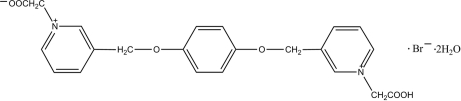


## Experimental

### 

#### Crystal data

C_22_H_21_N_2_O_6_^+^·Br^−^·2H_2_O
*M* *_r_* = 525.35
Monoclinic, 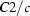

*a* = 20.605 (4) Å
*b* = 7.9612 (12) Å
*c* = 15.233 (4) Å
β = 113.845 (16)°
*V* = 2285.6 (8) Å^3^
*Z* = 4
Mo *K*α radiation
μ = 1.85 mm^−1^
*T* = 293 K
0.49 × 0.43 × 0.36 mm

#### Data collection

Bruker SMART CCD area-detector diffractometer
Absorption correction: multi-scan (*SADABS*; Sheldrick, 2004 ▶) *T*_min_ = 0.464, *T*_max_ = 0.556
2537 measured reflections
2009 independent reflections
1520 reflections with *I* > 2σ(*I*)
*R*_int_ = 0.047

#### Refinement

*R*[*F* ^2^ > 2σ(*F* ^2^)] = 0.045
*wR*(*F* ^2^) = 0.090
*S* = 1.09
2009 reflections
150 parameters
H-atom parameters constrained
Δρ_max_ = 0.52 e Å^−3^
Δρ_min_ = −0.33 e Å^−3^

### 

Data collection: *SMART* (Bruker, 2001 ▶); cell refinement: *SAINT* (Bruker, 2001 ▶); data reduction: *SAINT*; program(s) used to solve structure: *SHELXS97* (Sheldrick, 2008 ▶); program(s) used to refine structure: *SHELXL97* (Sheldrick, 2008 ▶); molecular graphics: *SHELXTL* (Sheldrick, 2008 ▶); software used to prepare material for publication: *SHELXTL* and local programs.

## Supplementary Material

Crystal structure: contains datablocks I, global. DOI: 10.1107/S1600536810037748/pb2040sup1.cif

Structure factors: contains datablocks I. DOI: 10.1107/S1600536810037748/pb2040Isup2.hkl

Additional supplementary materials:  crystallographic information; 3D view; checkCIF report

## Figures and Tables

**Table 1:** Hydrogen-bond geometry (Å, °)

*D*—H⋯*A*	*D*—H	H⋯*A*	*D*⋯*A*	*D*—H⋯*A*
O1—H1⋯O1^i^	0.82	1.65	2.459 (5)	168
O4—H4*B*⋯Br1	0.85	2.72	3.496 (3)	152
O4—H4*C*⋯O2^ii^	0.85	2.25	3.040 (4)	155

Symmetry codes: (i) 
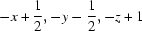
; (ii) 
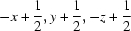
.

**Fig. 1.**
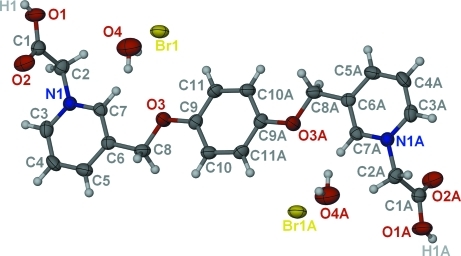
Ellipsoid plot.

**Fig. 2.**
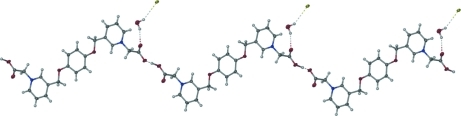
A portion of the infinite chain of the title compound viewed along the a direction, with atom labels of 30% probability displacement ellipsoids. Hydrogen bonds are displayed with dashed lines.

## supplementary crystallographic information

### Comment

The design and synthesis of substrates for the ultimate preparation of supramolecular species has received much attention in recent years. Double betaines are a class of zwitterionic compounds possessing pairs of carboxylate groups and quaternary ammonium or pyridinium moieties. The carboxylate group is basic, so betaines are good proton acceptors that easily form complexes with Bronsted acids.

The synthesis and crystal structure of 1:2 salt of 1,4-bis(3-picolyloxyl)benzene-*N*,*N*^'^– diacetic acid with HBr has been reported, here we will describe the preparation and structure of the 1:1 salt.

In the crystal structure of the title compound, the phenylene ring of the title double betaine is located at an inversion center, making a dihedra angle of 1.2 degree. Pairs of the betaine molecules are bridged by protons to form strong and symmetrical O···O hydrogen bonds, leading to an infinite chain. The bromide ion is connected to the betaine molecule through hydrogen bonding at O1W—H1WB···Br1 152.2 °, O1W···Br1 3.491 (4) Å, O1W—H1WA···O2 3.037 (4) Å,O1W···O2 154.5 °(Fig. 1).

### Experimental

1,4-bis(3-Picolyloxyl)benzene (2.92 g, 10 mmol) was dissolved in methanol (30 ml) to give a light yellow solution, to which ethyl bromoacetate (3 ml, 27 mmol, Aldrich) was added. The resulting solution was refluxed for 3 days. After the methanol was removed by rotary evaporation under reduced pressure, hydrobromic acid (12.5 ml, 4.8% (*w*/*v*)) was added to the yellow residue. The mixture was refluxed for 24 h to give a yellow solution. Removal of solvent afforded a light yellow powdery product. Yield: 46%. It was re-crystallized in water to obtain suitable single crystals for X-ray analysis.

### Refinement

H atoms in water molecule were located in a difference map. Other H atoms were positioned geometrically and refined using a riding model with C—H = 0.95–0.99 Å and with *U*_iso_(H) = 1.2 *U*_eq_(C).

### Crystal data

**Table:**

C_22_H_21_N_2_O_6_^+^·Br^−^·2H_2_O	*F*(000) = 1080
*M**_r_* = 525.35	*D*_x_ = 1.527 Mg m^−^^3^
Monoclinic, *C*2/*c*	Mo *K*α radiation, λ = 0.71073 Å
*a* = 20.605 (4) Å	Cell parameters from 186 reflections
*b* = 7.9612 (12) Å	θ = 2.0–27.6°
*c* = 15.233 (4) Å	µ = 1.85 mm^−^^1^
β = 113.845 (16)°	*T* = 293 K
*V* = 2285.6 (8) Å^3^	Block, light yellow
*Z* = 4	0.49 × 0.43 × 0.36 mm

### Data collection

**Table:**

Bruker SMART CCD area-detector diffractometer	2009 independent reflections
Radiation source: fine-focus sealed tube	1520 reflections with *I* > 2σ(*I*)
graphite	*R*_int_ = 0.047
phi and ω scans	θ_max_ = 25.0°, θ_min_ = 2.2°
Absorption correction: multi-scan (*SADABS*; Sheldrick, 2004)	*h* = −1→24
*T*_min_ = 0.464, *T*_max_ = 0.556	*k* = −1→9
2537 measured reflections	*l* = −18→16

### Refinement

**Table:**

Refinement on *F*^2^	Primary atom site location: structure-invariant direct methods
Least-squares matrix: full	Secondary atom site location: difference Fourier map
*R*[*F*^2^ > 2σ(*F*^2^)] = 0.045	Hydrogen site location: inferred from neighbouring sites
*wR*(*F*^2^) = 0.090	H-atom parameters constrained
*S* = 1.09	*w* = 1/[σ^2^(*F*_o_^2^) + (0.0331*P*)^2^ + 1.7877*P*] where *P* = (*F*_o_^2^ + 2*F*_c_^2^)/3
2009 reflections	(Δ/σ)_max_ < 0.001
150 parameters	Δρ_max_ = 0.52 e Å^−^^3^
0 restraints	Δρ_min_ = −0.33 e Å^−^^3^

### Special details

**Table:**

Geometry. All e.s.d.'s (except the e.s.d. in the dihedral angle between two l.s. planes) are estimated using the full covariance matrix. The cell e.s.d.'s are taken into account individually in the estimation of e.s.d.'s in distances, angles and torsion angles; correlations between e.s.d.'s in cell parameters are only used when they are defined by crystal symmetry. An approximate (isotropic) treatment of cell e.s.d.'s is used for estimating e.s.d.'s involving l.s. planes.
Refinement. Refinement of *F*^2^ against ALL reflections. The weighted *R*-factor *wR* and goodness of fit *S* are based on *F*^2^, conventional *R*-factors *R* are based on *F*, with *F* set to zero for negative *F*^2^. The threshold expression of *F*^2^ > σ(*F*^2^) is used only for calculating *R*-factors(gt) *etc*. and is not relevant to the choice of reflections for refinement. *R*-factors based on *F*^2^ are statistically about twice as large as those based on *F*, and *R*- factors based on ALL data will be even larger.

### Fractional atomic coordinates and isotropic or equivalent isotropic displacement parameters (Å^2^)

**Table:**

	*x*	*y*	*z*	*U*_iso_*/*U*_eq_	Occ. (<1)
Br1	0.0000	0.04115 (8)	0.2500	0.0454 (2)	
O1	0.20052 (13)	−0.2950 (4)	0.42592 (16)	0.0527 (7)	
H1	0.2364	−0.2608	0.4703	0.079*	0.50
O2	0.26011 (13)	−0.2308 (4)	0.33644 (16)	0.0519 (7)	
O3	0.05771 (12)	0.1842 (3)	0.05634 (16)	0.0410 (6)	
N1	0.14157 (13)	−0.2645 (3)	0.16985 (17)	0.0307 (6)	
C1	0.20678 (19)	−0.2737 (4)	0.3465 (2)	0.0351 (8)	
C2	0.13832 (18)	−0.3130 (5)	0.2610 (2)	0.0366 (8)	
H2A	0.1288	−0.4324	0.2600	0.044*	
H2B	0.0995	−0.2538	0.2679	0.044*	
C3	0.17237 (18)	−0.3677 (5)	0.1291 (2)	0.0398 (9)	
H3A	0.1902	−0.4708	0.1572	0.048*	
C4	0.17744 (18)	−0.3203 (5)	0.0454 (3)	0.0454 (10)	
H4A	0.1982	−0.3922	0.0161	0.054*	
C5	0.15208 (18)	−0.1677 (5)	0.0053 (2)	0.0410 (9)	
H5A	0.1561	−0.1351	−0.0509	0.049*	
C6	0.12032 (16)	−0.0615 (4)	0.0482 (2)	0.0304 (8)	
C7	0.11602 (16)	−0.1142 (4)	0.1314 (2)	0.0303 (8)	
H7A	0.0951	−0.0448	0.1617	0.036*	
C8	0.09107 (19)	0.1032 (4)	0.0026 (2)	0.0367 (8)	
H8A	0.1291	0.1732	0.0008	0.044*	
H8B	0.0569	0.0855	−0.0628	0.044*	
C9	0.02970 (17)	0.3417 (4)	0.0252 (2)	0.0327 (8)	
C10	0.02951 (18)	0.4206 (4)	−0.0558 (2)	0.0368 (9)	
H10A	0.0492	0.3675	−0.0937	0.044*	
C11	−0.00005 (18)	0.4215 (4)	0.0802 (2)	0.0367 (9)	
H11A	−0.0003	0.3684	0.1344	0.044*	
O4	0.15569 (16)	0.2619 (4)	0.28766 (19)	0.0738 (9)	
H4B	0.1174	0.2082	0.2578	0.111*	
H4C	0.1662	0.2720	0.2395	0.111*	

### Atomic displacement parameters (Å^2^)

**Table:**

	*U*^11^	*U*^22^	*U*^33^	*U*^12^	*U*^13^	*U*^23^
Br1	0.0537 (4)	0.0565 (4)	0.0345 (3)	0.000	0.0266 (2)	0.000
O1	0.0496 (16)	0.081 (2)	0.0317 (13)	−0.0026 (15)	0.0210 (12)	−0.0007 (13)
O2	0.0408 (15)	0.075 (2)	0.0394 (14)	−0.0151 (14)	0.0154 (12)	0.0005 (13)
O3	0.0565 (16)	0.0336 (15)	0.0416 (13)	0.0120 (12)	0.0289 (12)	0.0107 (11)
N1	0.0294 (15)	0.0312 (17)	0.0302 (14)	0.0004 (13)	0.0108 (12)	−0.0002 (13)
C1	0.039 (2)	0.032 (2)	0.0343 (18)	0.0022 (17)	0.0150 (16)	0.0015 (16)
C2	0.037 (2)	0.035 (2)	0.0370 (19)	−0.0008 (17)	0.0145 (16)	0.0067 (16)
C3	0.038 (2)	0.033 (2)	0.043 (2)	0.0067 (17)	0.0109 (17)	0.0006 (18)
C4	0.045 (2)	0.049 (3)	0.045 (2)	0.014 (2)	0.0218 (19)	−0.009 (2)
C5	0.044 (2)	0.051 (3)	0.0335 (18)	0.0026 (19)	0.0206 (17)	−0.0030 (18)
C6	0.0294 (17)	0.032 (2)	0.0280 (16)	−0.0034 (16)	0.0100 (14)	−0.0026 (16)
C7	0.0305 (18)	0.0292 (19)	0.0310 (16)	−0.0007 (16)	0.0122 (15)	−0.0046 (15)
C8	0.048 (2)	0.037 (2)	0.0305 (17)	0.0007 (18)	0.0209 (16)	0.0008 (16)
C9	0.0351 (19)	0.029 (2)	0.0335 (17)	−0.0007 (17)	0.0129 (15)	0.0052 (16)
C10	0.048 (2)	0.036 (2)	0.0313 (17)	0.0031 (17)	0.0207 (16)	0.0016 (16)
C11	0.046 (2)	0.036 (2)	0.0317 (17)	0.0028 (17)	0.0192 (16)	0.0097 (15)
O4	0.076 (2)	0.094 (2)	0.0528 (17)	−0.0131 (19)	0.0270 (16)	−0.0071 (17)

### Geometric parameters (Å, °)

**Table:**

Br1—Br1	0.0000 (12)	C5—C6	1.385 (4)
O1—C1	1.279 (4)	C5—H5A	0.9300
O1—H1	0.8200	C6—C7	1.372 (4)
O2—C1	1.218 (4)	C6—C8	1.492 (5)
O3—C9	1.382 (4)	C7—H7A	0.9300
O3—C8	1.419 (4)	C8—H8A	0.9700
N1—C3	1.335 (4)	C8—H8B	0.9700
N1—C7	1.342 (4)	C9—C11	1.376 (4)
N1—C2	1.469 (4)	C9—C10	1.383 (4)
C1—C2	1.516 (5)	C10—C11^i^	1.380 (5)
C2—H2A	0.9700	C10—H10A	0.9300
C2—H2B	0.9700	C11—C10^i^	1.380 (5)
C3—C4	1.373 (5)	C11—H11A	0.9300
C3—H3A	0.9300	O4—H4B	0.8498
C4—C5	1.366 (5)	O4—H4C	0.8494
C4—H4A	0.9300		
			
C1—O1—H1	109.5	C7—C6—C5	118.0 (3)
C9—O3—C8	116.5 (2)	C7—C6—C8	122.3 (3)
C3—N1—C7	121.5 (3)	C5—C6—C8	119.7 (3)
C3—N1—C2	119.3 (3)	N1—C7—C6	121.0 (3)
C7—N1—C2	119.1 (3)	N1—C7—H7A	119.5
O2—C1—O1	126.7 (3)	C6—C7—H7A	119.5
O2—C1—C2	121.6 (3)	O3—C8—C6	109.2 (2)
O1—C1—C2	111.7 (3)	O3—C8—H8A	109.8
N1—C2—C1	112.0 (3)	C6—C8—H8A	109.8
N1—C2—H2A	109.2	O3—C8—H8B	109.8
C1—C2—H2A	109.2	C6—C8—H8B	109.8
N1—C2—H2B	109.2	H8A—C8—H8B	108.3
C1—C2—H2B	109.2	C11—C9—O3	115.9 (3)
H2A—C2—H2B	107.9	C11—C9—C10	119.4 (3)
N1—C3—C4	119.4 (3)	O3—C9—C10	124.6 (3)
N1—C3—H3A	120.3	C11^i^—C10—C9	119.7 (3)
C4—C3—H3A	120.3	C11^i^—C10—H10A	120.1
C5—C4—C3	120.1 (3)	C9—C10—H10A	120.1
C5—C4—H4A	119.9	C9—C11—C10^i^	120.8 (3)
C3—C4—H4A	119.9	C9—C11—H11A	119.6
C4—C5—C6	120.0 (3)	C10^i^—C11—H11A	119.6
C4—C5—H5A	120.0	H4B—O4—H4C	95.3
C6—C5—H5A	120.0		
			
C3—N1—C2—C1	82.2 (4)	C5—C6—C7—N1	−0.3 (5)
C7—N1—C2—C1	−95.1 (3)	C8—C6—C7—N1	178.7 (3)
O2—C1—C2—N1	−10.1 (5)	C9—O3—C8—C6	177.9 (3)
O1—C1—C2—N1	171.3 (3)	C7—C6—C8—O3	−2.4 (4)
C7—N1—C3—C4	−0.7 (5)	C5—C6—C8—O3	176.5 (3)
C2—N1—C3—C4	−178.0 (3)	C8—O3—C9—C11	−177.6 (3)
N1—C3—C4—C5	1.0 (5)	C8—O3—C9—C10	2.8 (5)
C3—C4—C5—C6	−0.9 (5)	C11—C9—C10—C11^i^	0.5 (6)
C4—C5—C6—C7	0.5 (5)	O3—C9—C10—C11^i^	−180.0 (3)
C4—C5—C6—C8	−178.5 (3)	O3—C9—C11—C10^i^	179.9 (3)
C3—N1—C7—C6	0.4 (5)	C10—C9—C11—C10^i^	−0.5 (6)
C2—N1—C7—C6	177.6 (3)		

Symmetry codes:  (i) −*x*, −*y*+1, −*z*.

### Hydrogen-bond geometry (Å, °)

**Table:**

*D*—H···*A*	*D*—H	H···*A*	*D*···*A*	*D*—H···*A*
O1—H1···O1^ii^	0.82	1.65	2.459 (5)	168
O4—H4B···Br1	0.85	2.72	3.496 (3)	152
O4—H4C···O2^iii^	0.85	2.25	3.040 (4)	155

Symmetry codes: (ii) −*x*+1/2, −*y*−1/2, −*z*+1;  (iii) −*x*+1/2, *y*+1/2, −*z*+1/2.
